# Sudden hearing loss after intravitreal aflibercept injection

**DOI:** 10.5935/0004-2749.20210121

**Published:** 2025-08-21

**Authors:** Kemal Örnek, Emine Temel, Özkan Kocamış, Nazife Aşıkgarip

**Affiliations:** 1 Department of Ophthalmology, Kırşehir Ahi Evran University School of Medicine, Turkey; 2 Department of Ophthalmology, Kırşehir Ahi Evran Training and Research Hospital, Turkey

Dear Editor,

Intravitreal (IV) application of antiangiogenic drugs is an invasive procedure that requires following a safety protocol during application. Ocular side effects of anti-VEGF agents, such as subconjunctival hemorrhage, intraocular pressure elevation, vitreous hemorrhage, retinal detachment, and endophthalmitis, were addressed in a previous study^(1)^.

Serum levels of anti-VEGF agents were detected in the systemic circulation in patients treated with IV injections. The inhibition of circulating VEGF is associated with the reduction of nitric oxide (NO) production and prostaglandin-I2 (PG-I2), and the degeneration and death of vascular endothelial cells. Reduced NO and PG-I2 concentrations cause a peripheral vasoconstriction with consequent hypertension. Endothelial cell degeneration causes the exposure of phospholipids and extracellular matrix to pro-coagulant action in the lumen of the vessels, favoring thrombosis (myocardial infarction, transient ischemic attacks, etc.)^(2)^.

In this paper, we present a case of unilateral hearing loss after IV aflibercept injection in a patient with diabetic macular edema. A 62-year-old male patient had been treated and followed up for diabetic macular edema in the retina unit of our clinic for 2 years. He complained of a sudden hearing loss in his right ear 4 days after the last IV treatment. He had recently received an IV aflibercept injection treatment in his left eye.

The symptoms appeared on the fourth day after the patient received a consecutive dose of IV aflibercept (EYLEA, Bayer). He had tinnitus and hearing loss at the same time. He had no recent history of head trauma, upper respiratory tract infection, or ototoxic medication use. He had drug-controlled type 2 diabetes mellitus and no known medication allergy.

Otoscopic examination revealed normal external auditory canals and tympanic membranes on both sides. A pure tone audiogram demonstrated a profound hearing loss in his right ear (Figure 1) with a normal tympanogram. A magnetic resonance imaging scan confirmed the normal intracranial imagings; no abnormalities were found in the internal auditory meatus and cerebellopontine angle. Laboratory investigations, including complete blood count, full biochemistry, and systemic inflammatory markers, had normal results. The viral serological markers of cytomegalovirus, human immunodeficiency virus, hepatitis B and C, and syphilis serology were negative.

**Figure 1:**
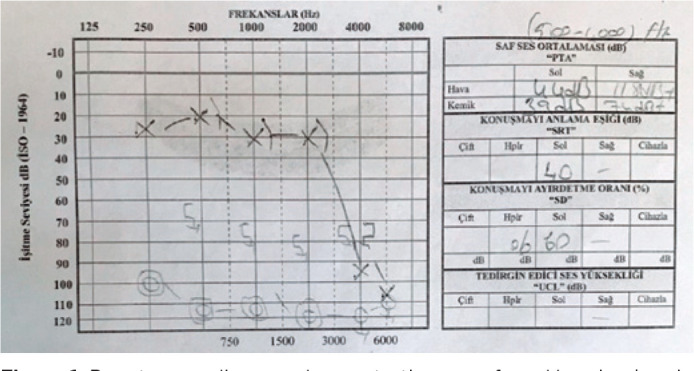
Pure tone audiogram demonstrating a profound hearing loss in the right ear with a normal tympanogram.

The patient started treatment after referral to the otorhinolaryngology department. After 10 days of treatment, no improvement in the hearing thresholds was observed.

Systemic effects that may be caused by anti-VEGF drugs include thromboembolic events, myocardial infarct, cerebrovascular events, and hypertension^(3)^. In a study by Nuzzi and Tridic, the systemic complications registered after IV bevacizumab injection included a case of nausea, an episode of chest pain with acute vision loss, and a case of acute blood hypertension^(4)^. An analysis of data from three randomized clinical trials that included a total of 859 patients with age-related macular degeneration revealed that IV ranibizumab was associated with an increased risk of cerebrovascular accidents when compared with sham treatment, whereas no apparent association was found between IV ranibizumab and myocardial infarction^(5)^.

Sudden sensorineural hearing loss is defined as a sudden hearing impairment with a hearing loss of >30 dB across three contiguous frequencies within a period of 72 hours. In 85% of cases, cochlear vascular microthrombosis has been hypothesized as a pathogenic mechanism in spite that no objective test is available for detecting the occlusion of such microcirculation^(6)^. Owing to the microvascular occlusive hypothesis, hypercoagulability (oral contraceptive use and pregnancy) or cardiovascular risk factors (hypertension, hyperlipidemia, diabetes, and smoking) can be investigated in patients with idiopathic sudden sensorineural hearing loss.

In our case, sudden hearing loss occurred on the fourth day after IV aflibercept injection. The patient consulted the department of otorhinolaryngology and received a diagnosis of total hearing loss in the right ear. Possible abnormalities in terms of hypercoagulability were investigated, and no abnormalities were detected. In this case, we propose that the sudden hearing loss might have been a consequence of the IV aflibercebt injection, considering the development of hearing loss in a short time after IV injection and previously reported microvascular complications due to anti-VEGF agents. Such a complication has not been reported in the literature before.

In conclusion, sudden hearing loss can occur after IV aflibercept injection. Further studies are needed to clarify the effects of IV injection of aflibercept on the auditory system, and the safety implications of the drug must be clarified.

## References

[1] Fasih U, Shaikh N, Rahman A, Sultan S, Fehmi MS, Shaikh A. (2013). A one-year follow-up study of ocular and systemic complications
of intravitreal injection of bevacizumab (Avastin). J Pak Med Assoc.

[2] Zarbin MA. (2018). Anti-VEGF agents and the risk of arteriothrombotic
events. Asia Pac J Ophthalmol (Phila).

[3] Csaky K, Do DV. (2009). Safety implications of vascular endothelial growth factor
blockade for subjects receiving intravitreal anti-vas cular endothelial
growth factor therapies. Am J Ophthalmol.

[4] Nuzzi R, Tridico F. (2015). Local and systemic complications after intravitreal
administration of anti-vascular endothelial growth factor agents in the
treatment of different ocular diseases: a five-year retrospective
study. Semin Ophthalmol.

[5] Ueta T, Yanagi Y, Tamaki Y, Yamaguchi T (2009). Cerebrovascular accidents in ranibizumab. Ophthalmology.

[6] Mom T, Gilain L, Avane P. (2008). [Cochlear ischemia: from fundamental data to clinical
hope]. Ann Otolaryngol Chir Cervicofac.

